# Do Wild Great Tits Avoid Exposure to Light at Night?

**DOI:** 10.1371/journal.pone.0157357

**Published:** 2016-06-29

**Authors:** Maaike de Jong, Jenny Q. Ouyang, Roy H. A. van Grunsven, Marcel E. Visser, Kamiel Spoelstra

**Affiliations:** 1 Department of Animal Ecology, Netherlands Institute of Ecology (NIOO-KNAW), Wageningen, The Netherlands; 2 Department of Biology, University of Nevada, Reno, Reno, NV, United States of America; 3 Plant Ecology and Nature Conservation Group, Wageningen University, Wageningen, The Netherlands; 4 Leibniz-Institute of Freshwater Ecology and Inland Fisheries, Müggelseedamm, Berlin, Germany; Università della Tuscia, ITALY

## Abstract

Studies of wild populations have provided important insights into the effects of artificial light at night on organisms, populations and ecosystems. However, in most studies the exact amount of light at night individuals are exposed to remains unknown. Individuals can potentially control their nighttime light exposure by seeking dark spots within illuminated areas. This uncertainty makes it difficult to attribute effects to a direct effect of light at night, or to indirect effects, e.g., via an effect of light at night on food availability. In this study, we aim to quantify the nocturnal light exposure of wild birds in a previously dark forest-edge habitat, experimentally illuminated with three different colors of street lighting, in comparison to a dark control. During two consecutive breeding seasons, we deployed male great tits (*Parus major*) with a light logger measuring light intensity every five minutes over a 24h period. We found that three males from pairs breeding in brightly illuminated nest boxes close to green and red lamp posts, were not exposed to more artificial light at night than males from pairs breeding further away. This suggests, based on our limited sample size, that these males could have been avoiding light at night by choosing a roosting place with a reduced light intensity. Therefore, effects of light at night previously reported for this species in our experimental set-up might be indirect. In contrast to urban areas where light is omnipresent, bird species in non-urban areas may evade exposure to nocturnal artificial light, thereby avoiding direct consequences of light at night.

## Introduction

Natural night-time darkness has disappeared across large parts of the world [1] as a result of anthropogenic lighting of the environment. Effects of artificial light at night on ecosystems are increasingly being studied over the past decade [2]. In order to assess latent and ecosystem-wide consequences, long-term experiments have been set up, e.g., in Germany [3], the United Kingdom [4] and the Netherlands [5]. These are starting to provide important insights into impacts of light at night on plant and animal populations, such as the suppression of flowering [4], the alteration of microbial communities [3] and the suppression or facilitation of mammal activity [5].

Effects of artificial light at night on an individual can be both direct and indirect. For example, a change in activity pattern could be a direct effect: the individual is exposed to the light, which affects its physiology and behavior (e.g., timing of reproductive physiology in birds [6]). But the same effect can also be indirect: light at night attracts prey species for the focal individual which consequently may change foraging activity (e.g., bats feeding on moths near lamps [7]).

Optimally, light intensities in experiments are chosen such that they are comparable to ‘real-life’ outdoor lighting situations. The light levels around light sources can be precisely measured but often the focal species is highly mobile. It is therefore difficult to know to how much light the studied individuals are actually exposed to, as these individuals are well able to move away from the light. Consequently, it becomes difficult to relate effects to experienced light levels, and effects observed in individuals that succeed to evade light at night may rather be indirect.

Few studies so far have measured nocturnal light levels as experienced by individual free-living animals. Dominoni et al. fitted rural and city blackbirds (*Turdus merula*) with light loggers and related individual light exposure to timing of daily activity [8] and subjective perception of day length [9]. Robert et al. [10] linked melatonin levels and timing of seasonal reproduction to exposure of individual tammar wallabies (*Macropus eugenii*) to light at night. Although in these cases the nighttime light exposure of focal individuals is known, these studies remain correlative since other anthropogenic factors that typically co-occur with artificial light, such as noise, cannot be excluded.

In an experimental set-up designed to assess the effects of artificial light at night of different colors on wild birds, we have so far observed that light at night can advance timing of egg laying [11] and increase corticosterone levels in the great tit (*Parus major*) [12]. It remains unclear whether these effects directly relate to increased light intensities at night, or whether these effects are more indirect. Information on the actual light exposure of individuals is essential in understanding its effects. We know precisely how the light intensity varies with distance to the lamps in this set-up, but we do not know whether birds avoid exposure to nocturnal artificial light as they can move away from the lamp posts at night and roost overnight in much darker places. Here, we assess to how much light individual great tits are exposed at night, and relate this to light levels at the location of their nest box.

## Methods

### Study area

We made use of a field site, Voorstonden, which is part of a long-term experiment in the Netherlands. In this experiment, previously dark natural areas are illuminated with white, green and red light, in comparison to a dark control area. For details about the set-up of this experiment and the characteristics of the light, see Spoelstra et al. [5]. The field site of this study is situated east of the Veluwe area (52°7’21” N; 6°7’7” E) and consists of deciduous and mixed forest edge habitat, with few natural cavities, and semi-natural grassland. Perpendicular to the forest edge, four transects have been set up, each with five, 4 m tall lamp posts. Each transect contains one of the four light treatments (white, green or red LED light, or dark control), and nine bird nest boxes at approximately 25 m distance from each other in a grid around the lamp posts. For details about the study on nest box breeding birds and a schematic overview of the field site, see de Jong et al. [11]. Light intensity at all nest box entrances was measured in four directions (upward, forward, to the left and to the right) with a calibrated illuminance meter (LMT B 360, LMT Lichtmesstechnik GmbH, Berlin, Germany). Averages of these four measurements are presented in Fig A in S1 Appendix for the nest boxes located within 30 m distance of the nearest lamp post.

### Light logger measurements

We measured the light intensities that free-ranging male great tits are exposed to at night with miniature light loggers (custom-made by Sigma Delta Technologies, Floreat, Western Australia, Australia) with a weight of ~0.95 g. including the harness. The light sensor (ISL29033, Intersil, USA) has a measuring range of 0.055 to 125 lux and a spectral sensitivity range from 300 to 700 nm. The sampling interval was set to five minutes and loggers were active for at least 24 hours. In 2014, between April 24 and May 16, and in 2015, between May 11 and 26, 30 birds total were equipped with a light logger. We caught the males of great tit pairs that were nesting in a nest box within 30 m of a lamp post or a dark control pole. During the second half of the egg incubation phase (eight nests in 2014, none in 2015), males were caught close to their nest box using a mist net and song play-back. During chick feeding phase (seven nests in 2014, 15 nests in 2015), males were caught in the nest box using a spring trap. Birds were ringed with a numbered aluminum ring and the light logger was attached to their back using a leg loop harness (photograph of bird with logger in Fig B in S1 Appendix). We aimed to evenly distribute the loggers over the four light treatments, but were dependent on the presence of great tit nests (see Table 1 for number of deployments in each treatment). To retrieve the loggers and collect the data, we tried to recapture birds during the same breeding season, using a spring trap or a mist net close to their nest box.

**Table 1:** Number of deployments and sample size.

	Dark	Green	Red	White	Total
**2014**					
Birds deployed	4	3	4	4	15
Birds caught back	2	3	4	3	12
Data available	0	2	2	2	6
**2015**					
Birds deployed	6	2	3	4	15
Birds caught back	2	1	3	2	8
Data available	1	1	3	2	7
**Total**					
Data available	1	3	5	4	13

Number of male great tits that were deployed with a logger, were caught back, and for which data are available in 2014 and 2015, and for both years, for the separate treatments and the totals. See also Fig D in S1 Appendix for the breeding locations of the light logger males.

### Light logger effects on nestling survival

When designing our light loggers, mass was the primary limiting factor. Male great tits weigh 18–19 g during the breeding season, which means that a light logger of ~0.95 g adds about 5% to their body weight. Although it is widely accepted that devices that add a maximum of 5% to the body mass of a bird do not significantly affect its behavior [13], a recent meta-analysis [14] showed that attaching devices to birds in general negatively affects most aspects of their behavior and ecology. To test whether deploying light loggers had a negative effect on parental care, we compared nestling survival (number of chicks that fledged / number of chicks that hatched; 1 = all chicks that hatched successfully fledged and 0 = no chicks that hatched successfully fledged), of the nests from which the male received a light logger (n = 14 in 2014, n = 15 in 2015) to those from which the male did not receive a light logger (n = 7 in 2014, n = 9 in 2015). All great tit nests at the field site were inspected regularly to assess the number of chicks that hatched and the number of chicks that fledged. We used a Mann-Whitney *U* test to compare nestling survival between nests with and without logger in each year, and found no differences (nestling survival in 2014: 0.93 ± 0.04 with logger and 0.95 ± 0.05 without logger (avg ± s.e.), Mann-Whitney U test: W = 55, p = 0.58; and in 2015: 0.82 ± 0.08 with and 0.78 ± 0.11 without logger (avg ± s.e.), Mann-Whitney U test: W = 57.5, p = 0.54). Nestling survival was generally high in this area and we found no difference between pairs with and without light loggers, thus we assume that the loggers did not cause behavioral differences that would affect reproductive success between the two groups of males.

### Data analysis

We validated the readings of the light loggers with a calibrated illuminance meter (LMT B 360) for all three light colours (see for details Fig C in S1 Appendix) and the original light logger measurements collected on birds were corrected for deviations from the illuminance meter measurements. We limited data analysis to the first 24 hours (for which we have data from all birds) and calculated the average light intensity received by the birds between two hours after sunset and two hours before sunrise (on average 4.5 hours). We excluded the hours after sunset and before sunrise, because earlier studies have shown that daily activity patterns are specifically affected by artificial light at night during these periods [15,16]. This way the measurements pertain to the resting period and are not confounded by shifts in activity patterns. We related the log of light intensity as measured on the bird, to the log of light intensity at the entrance of the nest box the pair was breeding in with a Spearman’s rank correlation test. Also, for the illuminated transects, we related both the logger and the nest box entrance average log light intensity to the distance between the nest box and the nearest lamp post, again using a Spearman’s rank correlation test.

### Ethics statement

Natuurmonumenten granted us permission to perform our experiment on their terrain; the natural area Voorstonden. The study was approved by the Animal Experimentation Committee of the Royal Netherlands Academy of Arts and Sciences and carried out under licence NIOO 10.07.

## Results

We were able to recapture 20 out of the 30 male great tits that were deployed with loggers, and we obtained light intensity data from 13 of them (see Table 1; in 2014 we obtained data from three loggers during egg incubation phase and three during chick feeding phase, in 2015 we obtained data from seven loggers during chick feeding). Seven birds either lost their light logger, or their logger failed to record any data. In Fig D in S1 Appendix, we show where the 13 males, from which we obtained data, have been breeding.

**Fig 1:**
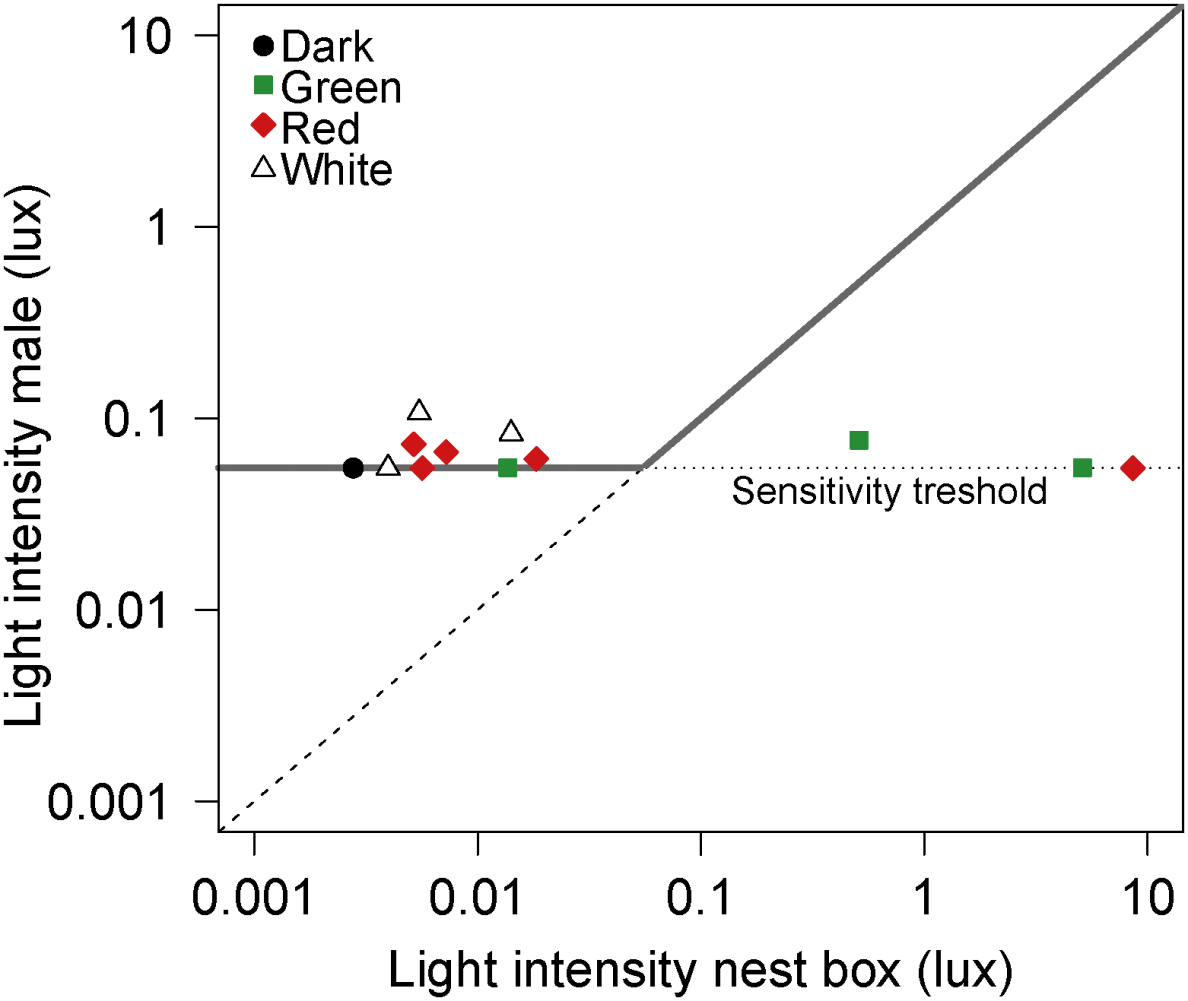
Night-time light exposure of male great tits. Average nocturnal light intensity (lux) that male great tits were exposed to (from two hours after sunset to two hours before sunrise), in relation to the light intensity (lux) measured at the nest box the pair was breeding in. The grey line indicates the expected light exposure if males would roost in the close surroundings of their nest box; this exposure remains level up to 0.055 lux because of the lower sensitivity threshold of the loggers (dotted line) and at higher intensities this is equal to light intensity measured at nest box entrance level (dashed line: light intensity male equals light intensity nest box). Filled black circle is the male with nest in the dark treatment, filled green squares are males with nest in the green treatment, filled red diamonds are males with nest in the red and open triangles are males in the white light treatment. Data from breeding seasons 2014 and 2015; error bars are not shown because standard errors are too small to be visible. Original light logger measurements were corrected to be comparable with the illuminance meter measurements in Fig A in S1 Appendix, see logger calibration data in Fig C in S1 Appendix.

The light intensity as recorded at the back of the male great tits did not change with increasing light intensity at the entrance of the nest box the pair was breeding in (Spearman’s rank correlation test: n = 13, rho = 0.15, p = 0.63; and see Fig 1). For the three males of pairs breeding in nest boxes closer than 10 m to the light posts, the average light intensity at the entrance of nest boxes is about 100 times higher than the average light level measured at the birds (respectively 6.79 lux and 0.062 lux, Fig 1). A correlation test for light intensity and distance to the nearest lamp post showed that light intensities at nest box entrance (in the illuminated transects) significantly decreased with distance to the nearest lamp post (Spearman’s rank; n = 20, rho = -0.76, p<0.001, Fig A in S1 Appendix). The same test for the male great tits nesting in the illuminated transects did not show a correlation between received light intensity and distance to the nearest lamp post (rho = 0.26, p = 0.41).

## Discussion

The light levels experienced by the male great tits nesting in the direct surroundings of the green and red lamp posts are much lower than the light levels measured at the nest boxes. The reduced exposure to light is only possible when these three males do not roost in close vicinity of their nest box (Fig 1). This suggests that these males have been avoiding light exposure at night by choosing a roosting place with a reduced light intensity, e.g., behind a tree trunk, high up in a tree, or further away from their nest box. Also concealment offered by branches and leaves may reduce light intensities. It is unlikely that the low values measured on birds resulted from males roosting in another nest box because >95% of all nest boxes were occupied by breeding pairs of great tits or other species. The low light levels measured may also originate from a possible habit of roosting further away from the nest box, however, males’ night roosting locations located with radio telemetry were usually within 10 m of their nest box location (JQ Ouyang, unpublished data). However, within this area darker places are still available.

Males from pairs breeding close to the lamp posts were not exposed to more light than males from pairs nesting further away, thus the breeding pair’s choice of nest location, in this area, does not influence nocturnal light exposure. Unfortunately, we had no measurements of males breeding close to white light and only one measurement of a male in the dark control treatment, but the fact that this male was exposed to a light intensity not different from those in the illuminated treatments (Fig 1) supports our conclusion. The possibility of birds avoiding light exposure at night was already touched upon by Dominoni et al. [8]; although the urban blackbirds in their study were exposed to higher light intensity at night than rural conspecifics, this intensity was at least 20-fold lower than the light intensity measurable in a 30 m radius from a common street lamp in the urban sites.

Although we have shown that nestling survival in nests of males that were deployed with a light logger was not lower than other nests, we cannot exclude that initial stress by capture and restraint has influenced the behavior of the males [17,18]. However, for birds from which we obtained more than 24 hours of data, light levels in the second night did not differ from those measured in the first night. Therefore, it seems unlikely that the observed avoidance of light during the first night results from an initial stress response.

The elevated corticosterone levels previously found in great tits nesting in the illuminated areas in this project could be a direct consequence of the light at night in the form of sleep disturbance [19], restlessness or alterations in circadian rhythms. Alternatively, these elevated levels could indirectly result from increased metabolism due to increased food availability and/or feeding rates, as discussed in Ouyang et al. [12]. The data we present here imply that the reported physiological changes may well be an indirect effect. Similarly, the advancement in lay date of great tits, as discussed in de Jong et al. [11], could be directly caused by a changed perception of day length, or, more likely in the light of the data presented here, could be related to a change in (timing of) abundance of prey species as a result of the artificial light at night.

The lower sensitivity boundary of our light loggers is 0.055 lux. This allows us to compare light levels in the direct surroundings of the lamp posts with levels that occur further away from them. However, light levels around 25 m distance from the lamps cannot be distinguished from background light levels as measured in the dark transects (see Fig A in S1 Appendix). Our measurements of light levels are done at only one field site, and the sample size is relatively low; we were able to obtain data from 13 males. The findings presented here, suggest the presence of indirect pathways of effects of nocturnal illumination, but more measurements are needed for a conclusive statement of how different light spectra affect behavior in free-living songbirds.

In rural and (semi-)natural areas, such as our study area, illumination is most often a linear structure like lighting along a road, with ample dark places around where birds can escape direct light exposure. In our experimental set-up, birds can use this possibility; male great tits seem to avoid artificial light at night. In this perspective, rural and (semi-) natural areas differ fundamentally from urban areas where light levels are not only higher but dark places needed to avoid exposure are less easy to find. The blackbirds in urban areas studied by Dominoni et al. are exposed to a generally higher light intensity, but also a higher variability, compared to rural birds [8]. Likewise, Robert et al. show that wallabies experience orders of magnitude more light at night in an urban compared to a natural area [10]. Differences in habitat structure and availability of dark areas between urban and non-urban environments thus result in different nocturnal exposure of birds and effects demonstrated in urban areas may not be easily extrapolated to more natural areas.

## Supporting Information

S1 Appendix Supporting information on light levels at study site, light logger deployment, validation of light loggers and breeding locations. Fig A: Light levels measured at study site. Fig B: Great tit deployment with light logger. Fig C: Validation of light loggers with illuminance meter. Fig D: Breeding locations of light logger males.
(DOCX)
