# Supplementary material for: Do Wild Great Tits Avoid Exposure to Light at Night?
Source: PLoS One. 2016 Jun 29;11(6):e0157357. doi: 10.1371/journal.pone.0157357 (PMC4927185; doi:10.1371/journal.pone.0157357)
Supplement: S1 Appendix — Fig A: Light levels measured at study site. Fig B: Great tit deployment with light logger. Fig C: Validation of light loggers with illuminance meter. Fig D: Breeding locations of light logger males. (DOCX) [file pone.0157357.s001.docx]

**Supporting Information**

de Jong M, Ouyang JQ, van Grunsven RHA, Visser ME, Spoelstra K. Do wild great tits avoid exposure to light at night?

**S1 Appendix. Supporting information on light levels at study site, light logger deployment, validation of light loggers and breeding locations.**


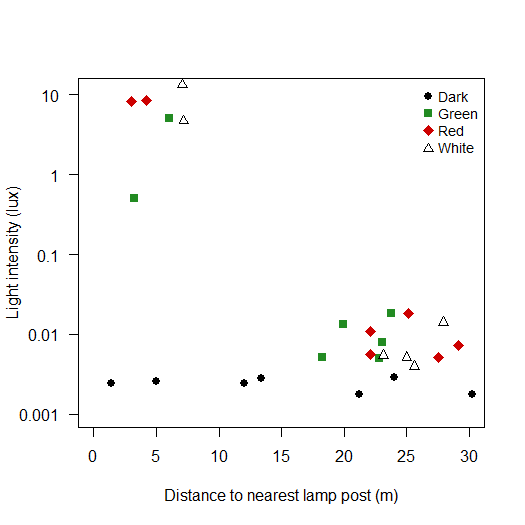


**Fig A. Light levels measured at study site.** Light intensity (lux) at nest box entrance level in relation to distance to the nearest lamp post (m) for all four light treatments; filled black circles are nest boxes in the dark treatment, filled green squares are nest boxes in the green treatment, filled red diamonds are nest boxes in the red and open triangles are nest boxes in the white light treatment. We present the average light intensity value of measurements, done with a calibrated illuminance meter, LMT B 360 (LMT Lichtmesstechnik GmbH, Berlin, Germany), in four directions (upward, forward, to the left and to the right) at each nest box entrance within 30 m distance of the nearest lamp post.


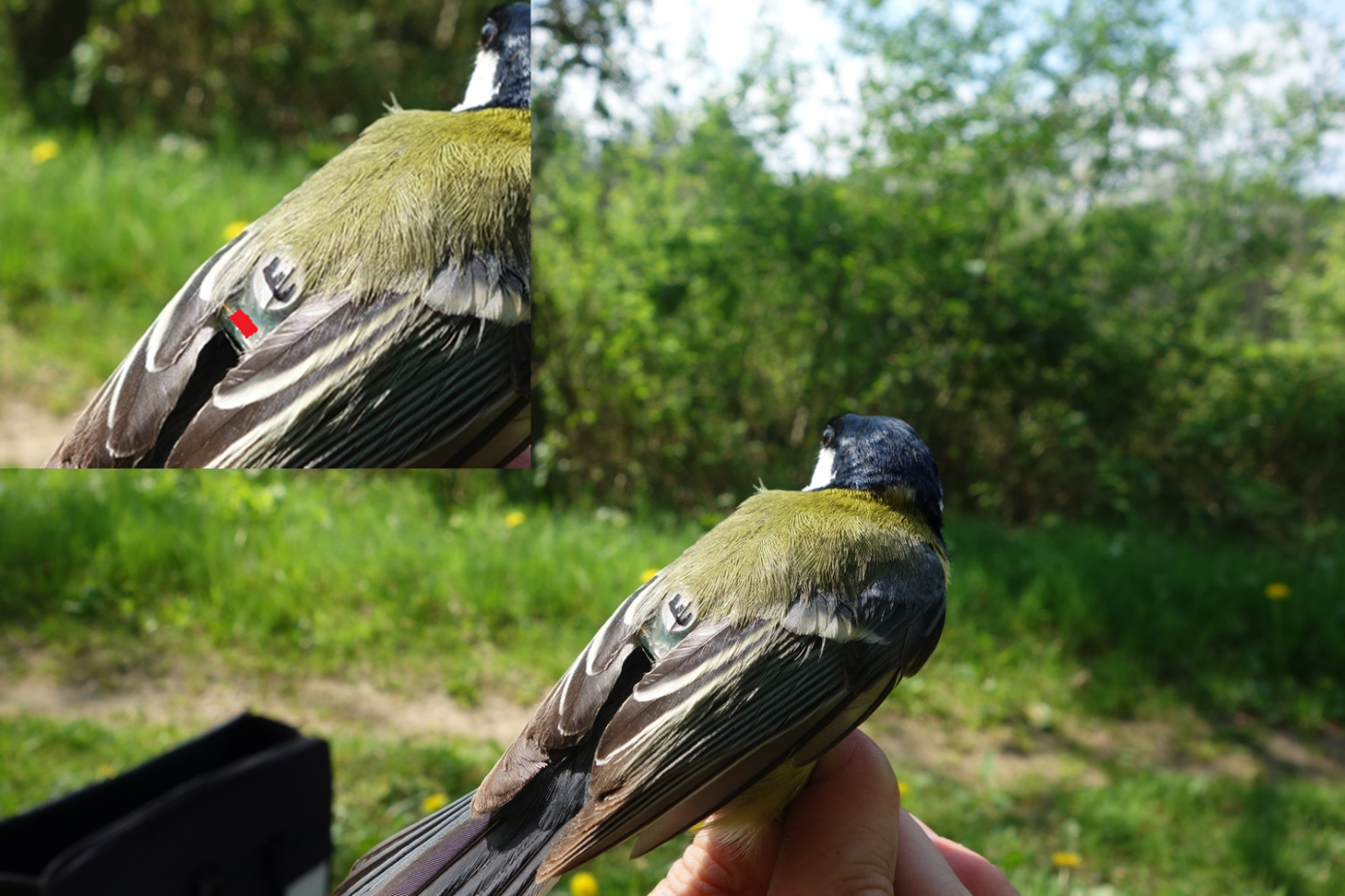


**Fig B. Great tit deployed with light logger.** Male great tit deployed with light logger ‘E’ (written on the round battery). The logger is attached at the back of the bird with a leg loop harness. The size of the leg loops was adjusted to match the bird during deployment. The light sensor is located just underneath the battery (indicated with red in the enlargement). Light logger measurements on captive great tits show that coverage of the sensor by feathers is not a potential problem; birds in a dark cage are exposed to light levels below the sensitivity threshold of the logger (≤0.055 lux), while birds in an illuminated cage are exposed to light levels equal to the levels measured there (measured on birds: average 1.17 ± s.e.0.28 and 2.92 ± 0.68; measured in the middle of the cages: ± 2 lux). Photograph made by J.Q. Ouyang on 24/04/2014 at field site Voorstonden, just before release of the bird with light logger.


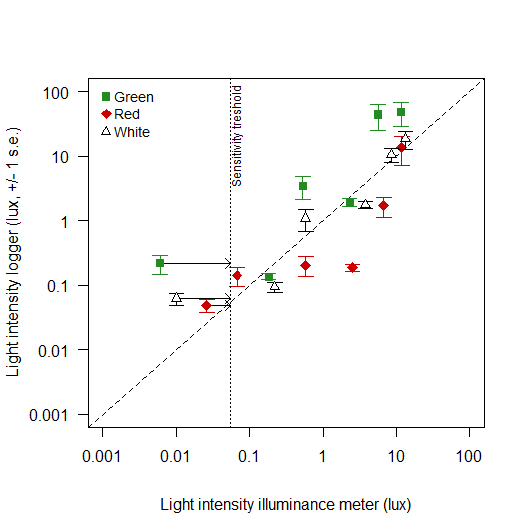


**Fig C. Validation of light loggers with illuminance meter.** The validation of the readings of the light loggers was done indoors, in a completely dark room, for each of the three light spectra (filled green squares for measurements under green light, filled red diamonds for red light and open triangles for measurements under white light). Measurements were done with a horizontally held, calibrated illuminance meter, LMT B 360 (LMT Lichtmesstechnik GmbH, Berlin, Germany), and at the same location with four light loggers, in three different directions (light sensor of logger facing towards the light source, away from the light source and horizontally with 90° angle to the light source). The averages of the light intensity in the three directions, measured by four light loggers (lux, ± 1 s.e.), in relation to the measurements of the illuminance meter (lux) are plotted. The sensitivity threshold of the light loggers is 0.055 lux (indicated with dotted line), which means that all measurements below this threshold are not reliable and are set to 0.055 lux (indicated with black arrows). Dashed line: light intensity logger equals light intensity illuminance meter.


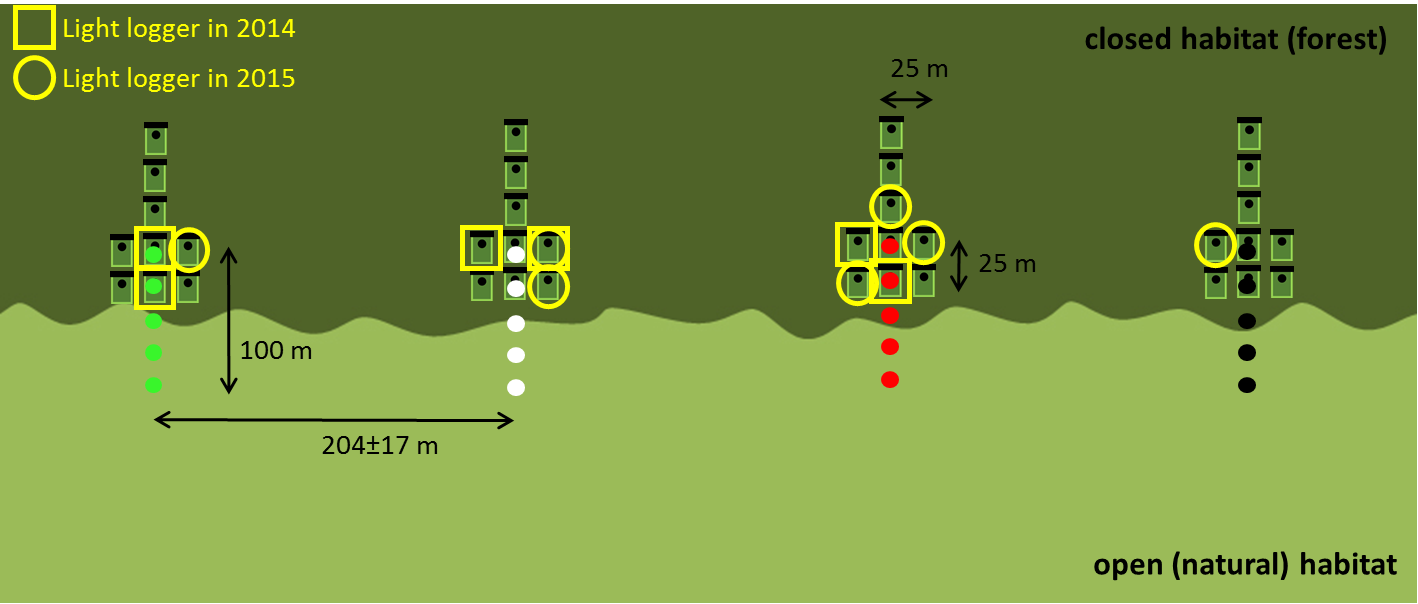


**Fig D. Breeding locations of light logger males.** Schematic overview of our study site, Voorstonden. Five, 4 m tall lamp posts (here green, white and red light respectively) and five dark control poles are placed in transects perpendicular to the forest edge. In each transect, nine nest boxes were attached to trees at 1.6 m height and at approximately 25 m distance from each other (dependent on the nearest tree). Orientation of the nest box opening was always towards the forest edge. Yellow squares indicate where the six males with light logger from which we obtained data in 2014 have been breeding. Yellow circles indicate where the seven males with light logger from which we obtained data in 2015 have been breeding. Note the nest box in the white transect from which we have data in 2014 as well as in 2015. Figure adapted from: de Jong M, Ouyang JQ, Da Silva A, van Grunsven RHA, Kempenaers B, Visser ME, Spoelstra K. Effects of nocturnal illumination on life-history decisions and fitness in two wild songbird species. Philosphical Trans R Soc B Biol Sci. 2015;370:20140128.
